# Resistance Response and Regulatory Mechanisms of Ciprofloxacin-Induced Resistant *Salmonella* Typhimurium Based on Comprehensive Transcriptomic and Metabolomic Analysis

**DOI:** 10.3390/antibiotics14080767

**Published:** 2025-07-29

**Authors:** Xiaohan Yang, Jinhua Chu, Lulu Huang, Muhammad Haris Raza Farhan, Mengyao Feng, Jiapeng Bai, Bangjuan Wang, Guyue Cheng

**Affiliations:** 1 National Reference Laboratory of Veterinary Drug Residues (HZAU) and MAO Key Laboratory for Detection of Veterinary Drug Residues, Huazhong Agricultural University, Wuhan 430070, China; yangxiaohan@webmail.hzau.edu.cn (X.Y.); chu@webmail.hzau.edu.cn (J.C.); huanglu@webmail.hzau.edu.cn (L.H.); Harisraza@webmail.hzau.edu.cn (M.H.R.F.); 2 MOA Laboratory for Risk Assessment of Quality and Safety of Livestock and Poultry Products, Huazhong Agricultural University, Wuhan 430070, China; fengmengyao@webmail.hzau.edu.cn (M.F.); 15128066762@webmail.hzau.edu.cn (J.B.); 15936571731@163.com (B.W.)

**Keywords:** *Salmonella* Typhimurium, drug resistance, ciprofloxacin, metabolomics, transcriptomics

## Abstract

Background: *Salmonella* infections pose a serious threat to both animal and human health worldwide. Notably, there is an increasing trend in the resistance of *Salmonella* to fluoroquinolones, the first-line drugs for clinical treatment. Methods: Utilizing *Salmonella* Typhimurium CICC 10420 as the test strain, ciprofloxacin was used for in vitro induction to develop the drug-resistant strain H1. Changes in the minimum inhibitory concentrations (MICs) of various antimicrobial agents were determined using the broth microdilution method. Transcriptomic and metabolomic analyses were conducted to investigate alterations in gene and metabolite expression. A combined drug susceptibility test was performed to evaluate the potential of exogenous metabolites to restore antibiotic susceptibility. Results: The MICs of strain H1 for ofloxacin and enrofloxacin increased by 128- and 256-fold, respectively, and the strain also exhibited resistance to ceftriaxone, ampicillin, and tetracycline. A single-point mutation of Glu469Asp in the GyrB was detected in strain H1. Integrated multi-omics analysis showed significant differences in gene and metabolite expression across multiple pathways, including two-component systems, ABC transporters, pentose phosphate pathway, purine metabolism, glyoxylate and dicarboxylate metabolism, amino sugar and nucleotide sugar metabolism, pantothenate and coenzyme A biosynthesis, pyrimidine metabolism, arginine and proline biosynthesis, and glutathione metabolism. Notably, the addition of exogenous glutamine, in combination with tetracycline, significantly reduced the resistance of strain H1 to tetracycline. Conclusion: Ciprofloxacin-induced *Salmonella* resistance involves both target site mutations and extensive reprogramming of the metabolic network. Exogenous metabolite supplementation presents a promising strategy for reversing resistance and enhancing antibiotic efficacy.

## 1. Introduction

*Salmonella* is a prevalent foodborne pathogen that can infect humans through the consumption of contaminated animal-derived products [1]. Research has demonstrated a significant genetic correlation between *Salmonella* isolates from human intestinal infections and those of avian origin, emphasizing the critical link between human *Salmonella* infections and the food supply chain [2]. Globally, non-typhoidal *Salmonella* causes approximately 95 million cases of foodborne diseases and 150,000 deaths each year [3]. *Salmonella* Typhimurium is one of the most common serotypes of non-typhoidal *Salmonella* and is frequently detected in livestock, poultry, and animal products across several countries [4]. Currently, fluoroquinolones are among the first-line drugs of choice for treating *Salmonella* infections. However, 29.2% of *S*. Typhimurium isolated from human patients with diarrhea and clinically suspected *Salmonella* infections are resistant to the first-line treatment drug ciprofloxacin [5]. The widespread prevalence of fluoroquinolone-resistant *Salmonella* has led the World Health Organization (WHO) to classify these resistant bacteria as one of the priority pathogens [6].

Mechanisms of bacterial resistance to quinolones include mutations in the *gyrA*, *gyrB*, *parC*, and *parE* genes within the quinolone resistance determining region (QRDR), as well as the expression of plasmid-mediated quinolone resistance (PMQR) genes [7]. Studies have shown that a single mutation in GyrA, which leads to the substitution of the proline (Pro) residue at position 864 with serine (Ser), may be closely related to fluoroquinolone resistance [8]. Analysis of QRDR mutations in *Salmonella* enterica isolates from around the world revealed that single mutations were prevalent at position 83 or 87 in GyrA [9]. However, mutations in the GyrB and ParE subunits were rarely observed in *Salmonella enterica* isolates [9]. Under antimicrobial pressure, the resistance developed in bacteria is not only attributed to the drug–target interactions but also involves changes that occur due to microbiota perturbations, metabolic processes, or changes in bacterial virulence [10]. Therefore, understanding the antibiotic-induced changes in the bacterial metabolic processes, local metabolites, and the effects of these metabolites on resistance dynamics is crucial for mitigating the spread of resistance.

Currently, transcriptomics and metabolomics technologies are widely employed to explore bacterial mechanisms underlying drug resistance. The combined application of multi-omics technologies provides complementary and validating insights at both gene and metabolite levels [11]. Recent studies have increasingly focused on the relationship between the metabolic profiles of drug-resistant *Salmonella* and antibiotic resistance mechanisms [11]. One study, using an untargeted metabolomics approach, explored the mechanisms of gentamicin-resistant *Salmonella* and found associations with disorders in central carbon metabolism and alterations in metabolic pathways, including those related to amino acids, nucleotides, vitamins, and cofactors [12]. Therefore, analyzing the expression of genes, proteins, and metabolites at different time points by employing omics techniques can provide a comprehensive understanding of bacterial responses to various antimicrobial drugs. However, due to a lack of transcriptomic and metabolomic studies on ciprofloxacin-resistant *Salmonella*, the associated changes in the metabolome and gene expression remain largely unexplored.

Compared with the short-term induction methods employed in previous studies, this research utilized ciprofloxacin to perform a continuous 58-day in vitro induction of *S*. Typhimurium. The dynamic transition of the bacterial strain from susceptibility to low-level resistance and subsequently to high-level resistance was systematically monitored. Following this, integrated transcriptomic and metabolomic analyses, both individually and in combination, were conducted to identify key genes and metabolic alterations associated with drug resistance, as well as to validate the influence of key metabolites on antibiotic interactions. This study provides a theoretical foundation for developing novel strategies to delay or mitigate drug resistance.

## 2. Results

### 2.1. Phenotypic Changes of Ciprofloxacin-Induced Resistant S. Typhimurium

**Figure 1:**
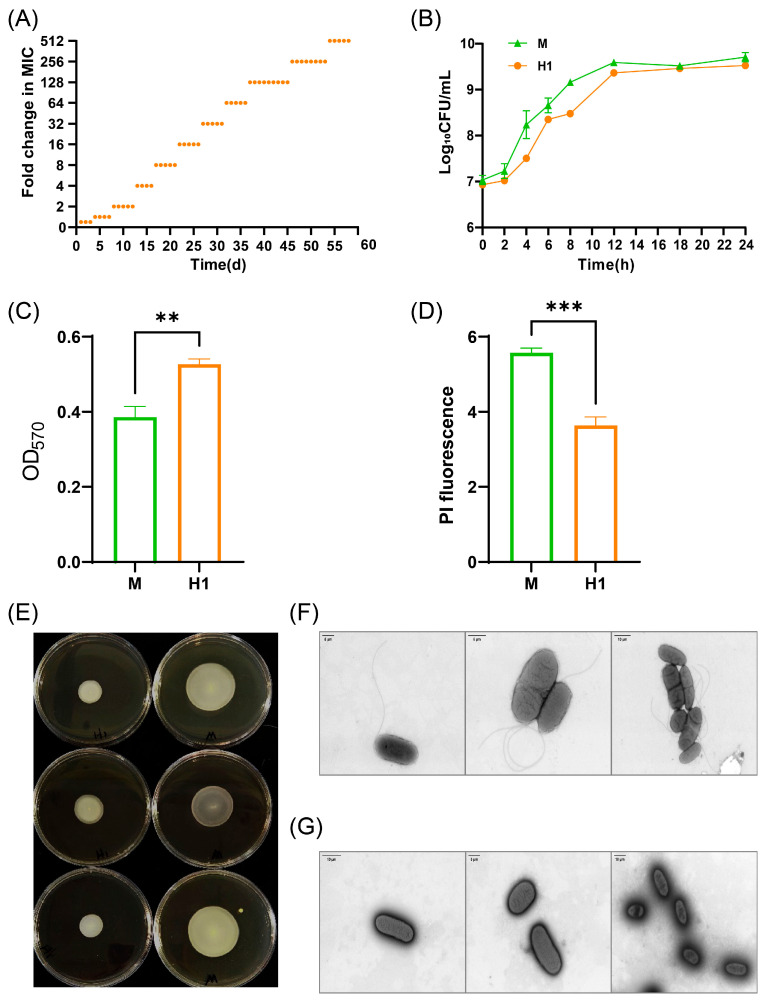
Phenotypic variation of strain H1. (**A**) MIC changes of strain H1 induced for 58 days. Each orange dot represents the fold change in MIC of the strain at different induction times. (**B**) Growth curves of strains M and H1. (**C**) Biofilm formation ability of strains M and H1. (**D**) Cell membrane permeability of strains M and H1. (**E**) Motility of strains M and H1: the left three dishes are strain H1, and the right three dishes are strain M. The colony formed by strain H1 on semi-solid agar was smaller than that formed by strain M, further indicating reduced motility. (**F**) Flagella of strain M observed by transmission electron microscopy. (**G**) Flagella of strain H1 observed by transmission electron microscopy. The flagellar morphology of strain H1 was altered, showing a sparse distribution compared to strain M. ** (*p* < 0.01), *** (*p* < 0.001).

The ciprofloxacin-resistant bacterium H1 (MIC = 2 μg/mL) was derived from the susceptible parental strain *S.* Typhimurium CICC10420 (M) through serial passage on agar plates with gradually increasing drug concentrations (Figure 1A). Upon further escalation of the drug concentration, bacterial growth ceased. Compared with the susceptible strain M, strain H1 showed increased MIC values for 16 drugs, in which the MICs of ciprofloxacin, ofloxacin, ceftriaxone, ampicillin, and tetracycline reached or exceeded the resistance cutoffs (Table 1). The MIC values of strain H1 for various antibiotics increased by at least two-fold, and in some cases, the actual fold change was even higher, indicating a substantial enhancement in drug resistance. After 5 days of passaging of strain H1 through drug-free medium, the resistance of strain H1 was stable. A mutation (A→C) at position 1398 in the *gyrB* gene was identified in strain H1.

No significant differences were observed in the growth curve of strain H1 compared to strain M (Figure 1B). However, strain H1 demonstrated enhanced biofilm formation and reduced cell membrane permeability, which may contribute to decreased susceptibility to multiple drugs (Figure 1C,D). Additionally, the motility of strain H1 was markedly diminished (Figure 1E), and transmission electron microscopy revealed that, unlike strain M, which possesses periplasmic flagella, most cells of strain H1 had lost their flagella (Figure 1F,G). This structural alteration may partially explain the observed decrease in motility (Figure 1E).

### 2.2. Transcriptome Profiling of Ciprofloxacin-Induced Resistant S. Typhimurium H1

**Figure 2:**
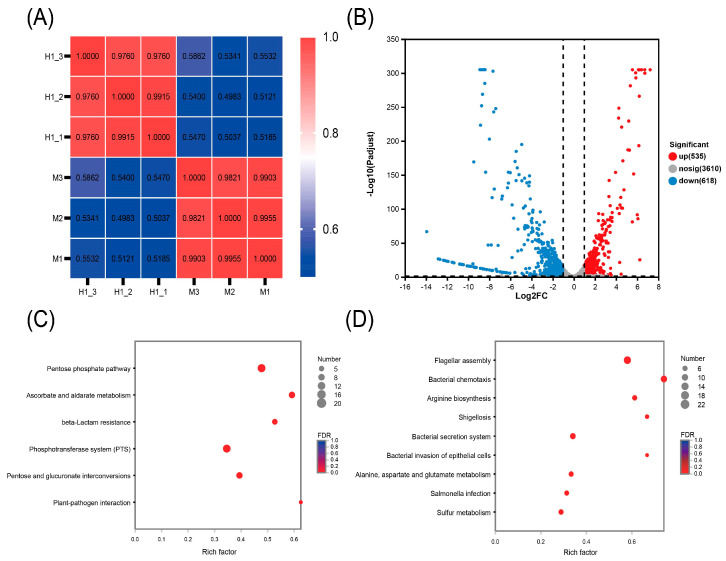
Transcriptomic changes of strain H1. (**A**) Heatmap illustrating the inter-sample correlation among strain H1 (*n* = 3) and strain M (*n* = 3). Correlation coefficients are represented between samples across the horizontal and vertical axes, with color gradients indicating the degree of correlation. (**B**) Volcano plot showing differentially expressed genes. Genes with an adjusted *p*-value < 0.05 and a fold change (FC) > 2 or <0.5 are defined as significantly differentially expressed. (**C**,**D**) KEGG-enriched metabolic pathways of the significantly upregulated (**C**) and downregulated genes (**D**) with enrichment rate on the horizontal axis and pathway names on the vertical axis. Circle size reflects the number of genes within each enriched pathway.

Transcriptional profiling of the parental susceptible strain M and the induced-resistant strain H1 was performed to elucidate the underlying molecular mechanisms of resistance. The heat map analysis revealed a strong correlation (close to 1) between the three biological replicates of both strains, indicating robust intragroup reproducibility (Figure 2A). A total of 535 genes were upregulated, while 618 genes were downregulated in strain H1 compared to strain M (Figure 2B).

Upregulated genes in strain H1 were primarily associated with the pentose phosphate pathway and β-lactam resistance (Figure 2C). Enhanced expression of genes in the pentose phosphate pathway can significantly boost cellular antioxidant capacity, thereby reducing oxidative damage caused by antibiotics and contributing to increased drug resistance [13]. The upregulation of β-lactam resistance genes suggests that strain H1 is developing mechanisms to counteract β-lactam antibiotics [14]. Collectively, the activation of these two pathways enhances bacterial survival under antibiotic stress, reinforcing the development of resistance. Downregulated genes were associated with flagellar assembly and bacterial chemotaxis (Figure 2D). These two processes are crucial for bacterial motility. Reduced expression of genes in these pathways may lead to diminished motility and an increased capacity for biofilm formation [15,16]. This implies that ciprofloxacin-resistant bacteria may conserve energy by reducing motility to better adapt to their environment, reflecting the metabolic cost of adaptation [17].

**Figure 3:**
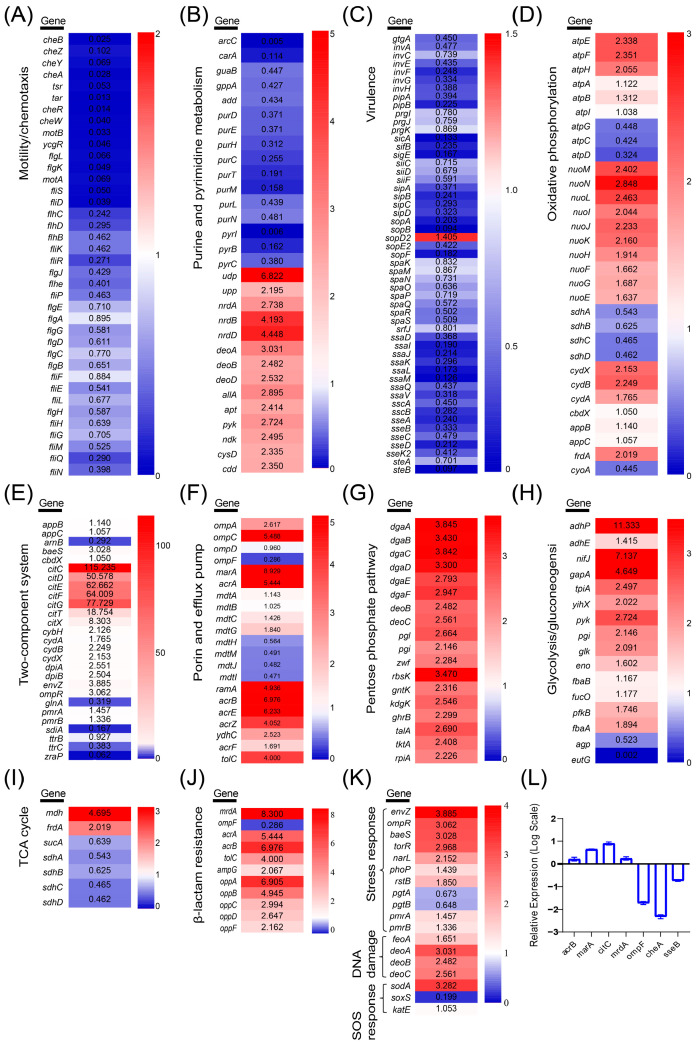
Differentially expressed genes in metabolic pathways. (**A**) Motility and chemotaxis. (**B**) Purine and pyrimidine metabolism. (**C**) Virulence. (**D**) Oxidative phosphorylation. (**E**) Two-component system. (**F**) Porin and efflux pump. (**G**) Pentose phosphate pathway. (**H**) Glycolysis/glycolysis. (**I**) Tricarboxylic acid cycle. (**J**) β-lactam resistance. (**K**) Stress response. For each gene in above pathway groups, the fold-change value was displayed within each box. Color intensity reflects the degree of up- or downregulation. (**L**) Relative transcriptional levels of seven genes in H1 determined by qRT-PCR.

All genes related to chemotaxis and flagellar assembly were downregulated in H1 (Figure 3A). Key genes involved in purine and pyrimidine metabolism (e.g., *ndk*, *nrdA*, *nrdB*, *nrdD*, *pyk*, *adk*, *udp*, *cdd*) were also upregulated, suggesting a potential compensatory response to drug stress (Figure 3B). Most virulence-related genes except sopD2 were downregulated in H1, with 36 genes significantly downregulated (Figure 3C). Both strains H1 and M displayed significant enrichment of DEGs in the two-component signaling system. Notably, *citC*, a key gene in citrate metabolism, was upregulated 115.235-fold, along with cytochrome oxidase genes (*cydA*, *cydB*, *cydX*) and the genes encoding for signaling systems (*envZ* and *ompR*) (Figure 3E). Upregulation was also noticed in outer membrane protein genes (*ompA* and *ompC*), while ompD and ompF were downregulated. Additionally, genes encoding efflux pump proteins (AcrA, AcrB, AcrE, AcrZ, TolC) and transcriptional regulators (MarA and RamA) were significantly upregulated (Figure 3F). Upregulation of all genes in the pentose phosphate pathway in H1 may indicate a compensatory response to ciprofloxacin-induced DNA damage (Figure 3G).

The expression of genes involved in central carbon metabolism, including glycolysis and the tricarboxylic acid (TCA) cycle, was also assessed (Figure 3H,I). Most glycolytic pathway genes were upregulated in H1, and the gene encoding malate dehydrogenase (*mdh*) was upregulated in the TCA cycle. The oxidative phosphorylation pathway supplies ATP to the body and provides the necessary energy. The expression of genes encoding the mitochondrial ATP synthase complex in H1, namely *atpE*, *atpF*, and *atpH*, as well as genes related to NADH dehydrogenase, namely *nuoM*, *nuoN*, *nuoL*, *nuoI*, *nuoJ*, and *nuoK*, significantly increased, whereas succinate dehydrogenase genes (*sdhA*, *sdhB*, *sdhC*, and *sdhD*) were downregulated (Figure 3D). Analysis of β-lactam resistance genes revealed a significant upregulation of *mrdA*, *acrA*, *acrB*, *tolC*, *mrdG*, *ampG*, *oppA*, *oppB*, *oppC*, *oppD*, and *oppF* (Figure 3J), potentially contributing to increased MIC values against ceftriaxone and ceftiofur (Table 2). Moreover, genes associated with stress response (*envZ*), SOS response (*sodA*, *katE*), and DNA damage repair (*feoA*, *deoA*, *deoB*, *deoC*) were also upregulated in H1 (Figure 3K). qRT-PCR confirmed the expression trends of seven genes (*acrB*, *marA*, *citC*, *mrdA*, *ompF*, *cheA*, *sseB*) in H1, consistent with transcriptomic results (Figure 3L).

The SNP analysis identified non-synonymous mutations in the exon region of H1 (Table 2). Previous studies have demonstrated that *gyrB* in H1 has undergone mutations, which can result in the overexpression of multidrug resistance efflux pumps [18]. In H1, multiple genes associated with the efflux pump are upregulated, leading to reduced sensitivity to various antibiotics. Additionally, a single mutation was detected in the *atpA* gene, which encodes ATP synthase, in strain H1. This gene is critical for cellular energy metabolism and influences the expression of multiple genes involved in the oxidative phosphorylation pathway. Furthermore, the *feoB* gene, encoding the iron ion transporter, has experienced double mutations. The Feo system facilitates efficient iron uptake, enabling bacteria to survive and reproduce in iron-limited environments, thereby enhancing bacterial virulence [19]. Notably, the expression of virulence-related genes in strain H1 was significantly downregulated.

### 2.3. Metabolomic Profiling of Ciprofloxacin-Induced Resistant S. Typhimurium H1

**Figure 4:**
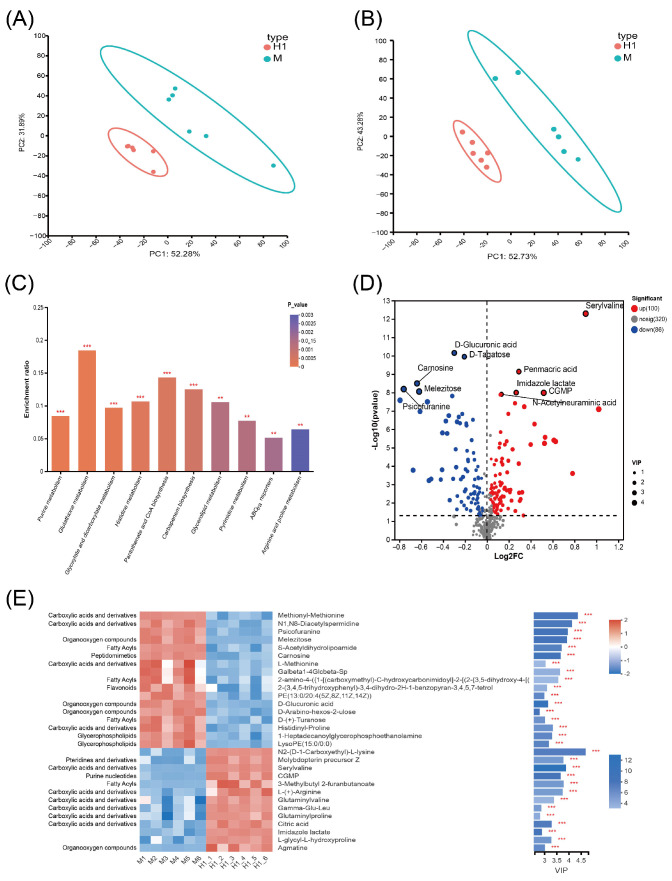
Metabolomic profiling changes of H1 and M. (**A**,**B**) PCA distribution plots in cationic (**A**) and anionic modes (**B**). (**C**) Top 10 differential metabolite enrichment KEGG pathways. The enrichment rate is proportional to the number of metabolites annotated to the pathway. The *p*-value size is shown by a color gradient. (**D**) Differential metabolite distribution volcano plot. The horizontal coordinate is the logarithmic value of FC of the metabolite between the two groups, and the vertical coordinate is the logarithmic value of the *p*-value, and the size of the dots in the plots indicates the VIP value. (**E**) Differential metabolite clustering heatmap (left) and VIP bar graph (right). The VIP bar graph reflects the size of the VIP value by the length of the bar, and the color depth of the bar reflects the size of the metabolite *p*-value. The darker the color, the smaller the *p*-value. ** or *** represents a *p*-value < 0.01 or <0.001, respectively.

The PCA plots illustrated inter-sample variability based on the distances between sample points, revealing clear distinctions between the resistant strain H1 and its parental strain M in both cationic and anionic ionization modes (Figure 4A,B). In these plots, the cumulative variance explained by PC1 and PC2 exceeded 80%, indicating that the first two principal components captured the majority of the variation in the dataset. Biological replicates of each group fell within the confidence ellipse, and quality control (QC) samples demonstrated reproducibility.

The top ten significantly different metabolic pathways (*p* < 0.05) enriched in H1 were purine metabolism, glutathione metabolism, acetate and dicarboxylate metabolism, histidine metabolism, pantothenic acid and coenzyme A biosynthesis, carbocyanine biosynthesis, glycerol ester metabolism, pyrimidine metabolism, ABC transporter, and arginine and proline metabolism (Figure 4C). Metabolites with VIP > 1 and *p*-value < 0.05 in H1 were regarded as significantly different metabolites. Notably, the majority of these significantly different metabolites in H1 were associated with amino acids. Among them, the significantly upregulated metabolites included N2-(D-1-Carboxyethyl)-L-lysine, serylvaline, L-(+)-arginine, glutaminylvaline, Gamma-Glu-Leu, glutaminylproline, and L-glycyl-L-hydroxyproline, whereas the significantly downregulated metabolites included methionyl-methionine, L-methionine and histidinyl-proline (Figure 4D,E). Arginine synthesis is closely tied to glutamine metabolism. The increase in glutamine metabolites can help bacteria maintain energy metabolism under antibiotic stress, enhance antioxidant capacity, and thereby increase resistance [20]. In the mechanism of resistance, changes in methionine metabolites can significantly influence bacterial susceptibility to antibiotics [21]. Upregulated amino acid metabolites may contribute to the repair of damaged cellular structures, whereas downregulated metabolites may be associated with reduced energy expenditure. These metabolic shifts collectively support bacterial survival in adverse conditions [22].

### 2.4. Integrated Analysis of Transcriptome and Metabolome

**Figure 5:**
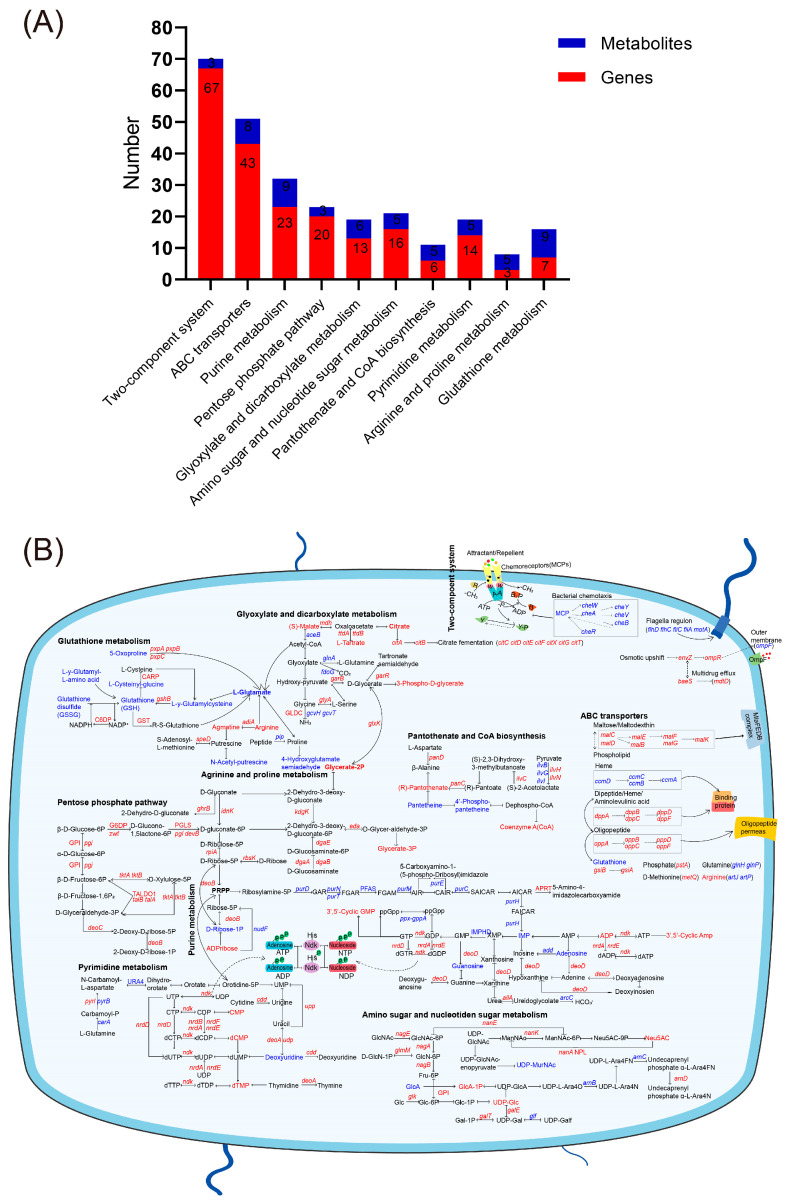
KEGG metabolic pathways co-enriched for both differential genes and metabolites. (**A**) Co-enrichment analysis of KEGG metabolic pathways involving both differential genes and metabolites. Metabolites with a VIP > 1 and *p*-value < 0.05 are considered significantly differentially expressed. The metabolic pathways associated with these metabolites were identified through KEGG database annotations. (**B**) Expression changes of genes and metabolites in the co-enriched KEGG metabolic pathway. Red color represents upregulated genes or metabolites, and blue color represents downregulated genes or metabolites.

Joint analysis of differential genes and metabolites revealed compatibility between genotypic and phenotypic results. KEGG pathways co-enriched for both differential genes and metabolites included the two-component system, ABC transporter, purine metabolism, pentose phosphate pathway, glyoxylate and dicarboxylic acid metabolism, amino sugar and nucleotide sugar metabolism, pantothenic acid and coenzyme A biosynthesis, pyrimidine metabolism, arginine and proline biosynthesis metabolism, and glutathione metabolism (Figure 5A).

The pentose phosphate pathway in strain H1 was significantly enriched with upregulated expression of genes such as pgi and zwf, promoting glucose-6-phosphate and NADPH production, respectively. The increase in NADPH can support the reduction of glutathione (GSH), enhance the antioxidant defense capacity of cells, and thereby increase drug resistance [23]. In the purine metabolism pathway, downstream metabolites ADP and cAMP in the ATP degradation pathway and cGMP in the GTP degradation pathway were upregulated, along with related regulatory genes *ndk*, *nrdD*, *nrdA*, and *nrdB*. The upregulation of these genes may be related to the energy metabolism reprogramming of bacteria under antibiotic stress, helping bacteria maintain ATP levels and thereby supporting the key resistance mechanisms [24]. However, adenine nucleoside, guanine nucleoside, and genes involved in inosine monophosphate (IMP) catabolism (*guaB* and *add*) were downregulated, possibly due to inhibition of the IMP de novo synthesis pathway (Figure 3B). Pyrimidine metabolism showed upregulation of downstream metabolites CMP, dCMP, dTMP, and related genes (*ndk*, *nrdD*, *nrdA*, *nrdB*). Amino sugar and nucleotide sugar metabolism pathways showed upregulation in N-acetylneuraminic acid (Neu5Ac), with transcriptomics revealing upregulation of genes (*nanA*, *nanE1*, and *nanM*) regulating this pathway. Levels of UDP-N-acetylmuramate (UDP-MurNAc) were reduced, and the downregulation of UDP-MurNAc may relate to the metabolic pathway wherein UDP-N-acetylglucosamine (UDP-GlcNAc) primarily contributes to Neu5Ac synthesis. Under the pressure of antibiotics, some downstream metabolites and gene expressions in the purine and pyrimidine metabolic pathways of bacteria are enhanced. This may indicate that bacteria activate the de novo synthesis pathways of purine and pyrimidine nucleotides to ensure an adequate supply. The de novo synthesis of purine nucleotides begins with 5-phosphoribose, which is progressively converted into inosine monophosphate (IMP), and subsequently into adenosine monophosphate (AMP) and guanosine monophosphate (GMP). The pyrimidine nucleotide synthesis pathway starts with carbamoyl phosphate, leading to the formation of orotate, which is then converted into uridine monophosphate (UMP), and further into cytidine triphosphate (CTP) and deoxythymidine monophosphate (dTMP) [25]. Co-analysis revealed that the development of ciprofloxacin resistance in bacteria necessitates compensation in purine and pyrimidine metabolism.

Multiple genes in the ABC transporter and the two-component system were altered in their expression, affecting bacterial motility and chemotaxis as well as the state of the cell membrane. Within the ABC transporter pathway, elevated levels of lysine and arginine were observed, alongside decreased levels of aspartate, glutamate, and glutathione. These changes may be related to the antioxidant responses of bacteria under antibiotic stress, helping bacteria reduce oxidative stress damage [24]. The glyoxylate and dicarboxylate metabolism showed increased levels of (S)-malate, citrate, L-tartrate, and 3-phospho-D-glycerate, alongside a decrease in L-glutamate. In glutathione metabolism, reduced levels of reduced glutathione (GSH) and oxidized glutathione (GSSG) indicated an antioxidant response under ciprofloxacin pressure. Arginine and proline biosynthesis showed reduced proline but increased arginine levels, suggesting supplementation from other metabolic pathways. Additionally, pantothenate and coenzyme A (CoA) biosynthesis was upregulated, with increased expression of the upstream gene (*panC*), providing essential nutrients for H1. These metabolic alterations not only impact energy metabolism but may also enhance bacterial antioxidant capacity and DNA repair mechanisms through the regulation of gene expression and metabolite levels, thereby reinforcing the drug resistance phenotype.

### 2.5. The Effects of Exogenous Metabolites on the Susceptibility of CIP-Resistance Bacterial

Activating bacterial metabolic processes offers a promising strategy for combating antibiotic resistance [26]. This study focused on drugs that exhibited a significant increase in minimum inhibitory concentration (MIC) multiples, such as ciprofloxacin, ceftriaxone, ampicillin, and tetracycline, as well as amino acid metabolic products that significantly changed in strain H1, including glutamine, reduced glutathione, glutamic acid, aspartic acid, methionine, and proline. The aim was to investigate their effects on the antibiotic resistance of strain H1. The checkerboard assay showed that the combination of glutamine with tetracycline yielded an FIC index of 1 (Table 3), indicating an additive effect without synergistic enhancement. The sub-inhibitory concentration of tetracycline was reduced two-fold when combined with glutamine. Other metabolic products combined with ciprofloxacin, ceftriaxone, ampicillin, or tetracycline either had no impact or even enhanced the strain’s resistance to ciprofloxacin. However, combinations of glutamine, GSH, glutamic acid, and aspartic acid with ampicillin and ceftriaxone, while showing no interaction effect, could reduce the MIC of these drugs by up to 1024-fold. This substantial decrease in MIC suggests that these compounds may indirectly enhance antibiotic efficacy through mechanisms such as efflux pump inhibition or reduction of β-lactamase activity.

## 3. Discussion

Multiple studies have reported mutations in the *gyrB* gene. For instance, the Asp426Asn mutation in *Escherichia coli* has been shown to confer quinolone resistance [27], while the Lys447Glu mutation confers resistance to nalidixic acid but retains susceptibility to ciprofloxacin [28]. In this study, we identified a Glu469Asp mutation in GyrB. This mutation has previously been observed in quinolone-resistant *Pseudomonas putida*, *Pseudomonas aeruginosa*, and *Haemophilus influenzae*, and has been associated with reduced susceptibility to quinolones [29,30,31], although it has not yet been reported in *Salmonella*. No mutations were detected in topoisomerases ParC and ParE. In this study, the induced strain H1 not only exhibits resistance to fluoroquinolone drugs, but also exceeds the resistance threshold for various other antibiotics. Therefore, the substitution of aspartic acid at position 469 in the *gyrB* gene of strain H1 may have broader implications for drug resistance. This mutation may reveal novel resistance mechanisms, contributing to a deeper understanding of the molecular basis of bacterial resistance. Clarifying the impact of this mutation can improve the accuracy of predicting bacterial drug resistance and thereby guide the rational use of antibiotics.

Bacterial motility is predominantly reliant on flagella or pili, and this capability plays a critical role in enabling bacteria to colonize specific sites, migrate toward more favorable environments, and evade adverse conditions [32]. KEGG pathway analysis demonstrated that genes associated with bacterial motility and flagellar assembly were significantly enriched; however, the expression of motility-related genes was uniformly downregulated. Strain H1 exhibited a marked reduction in motility on solid media, and transmission electron microscopy revealed that its flagellar formation capacity was diminished compared to the sensitive strain M. Given that flagellum-mediated motility serves as a key determinant for intestinal colonization in many organisms [33], the intestinal colonization ability of strain H1 following ciprofloxacin exposure may be compromised.

Bacterial biofilms confer antibiotic tolerance and resistance through diverse mechanisms [34]. In strain H1, the upregulation of the *ompA* gene, which is associated with bacterial adhesion and biofilm formation, promotes biofilm development and enhances resistance to multiple antibiotics [35]. The reduced expression of outer membrane porin OmpF decreases membrane permeability, thereby limiting intracellular drug accumulation [36]. This reduction in *ompF* expression in H1 leads to decreased cell membrane permeability and further elevates bacterial resistance. Additionally, cell membrane permeability is closely linked to multidrug efflux pumps, a primary mechanism of antibiotic resistance. In H1, the genes *marA*, *acrAB*, and *tolC*, which are involved in efflux pump regulation, are significantly upregulated [37]. Furthermore, downstream genes of the CitAB two-component system (*citC*, *citG*, *citF*, *citE*) and regulatory genes *narL* and *torR* exhibit marked upregulation, potentially mediating resistance by altering the expression of resistance determinants in H1 [38].

Metabolomics studies have shown that sarafloxacin-induced resistant *Salmonella* strains exhibit reduced levels of certain metabolites involved in purine/pyrimidine metabolism [11]. Similarly, in this study, both increased and decreased metabolites are enriched in purine/pyrimidine metabolic pathways, suggesting a strong association between purine metabolism and bacterial resistance. Research has also demonstrated that disrupting thymidylate synthesis can restore bacterial sensitivity to cephalosporins [39]. A reduction in dTMP content may increase antibiotic sensitivity by disturbing the balance between DNA repair and reactive oxygen species (ROS) production. The elevated dTMP levels observed in this study may be directly linked to the maintenance of drug resistance. The glutathione system serves as a key antioxidant defense mechanism in Gram-negative bacteria, and alterations in glutathione biosynthesis reflect changes in the bacterial redox state. Studies have demonstrated that bactericidal antibiotics can reduce the GSH/GSSG ratio in bacteria [40]. In this study, the glutathione metabolic pathway in strain H1 was significantly altered, indicating that the bacteria mounted an antioxidant response. Furthermore, studies have shown that both exogenous (environmental) and endogenous (biosynthetic) L-arginine promote biofilm formation by modulating c-di-GMP content and altering the expression of extracellular matrix structural components [41]. In this study, the increased arginine content was associated with enhanced biofilm formation and drug resistance. Additionally, exogenous methionine has been shown to promote intracellular accumulation of tigecycline by enhancing bacterial proton motive force, effectively re-sensitizing tet(X)-positive pathogens to the drug [21]. In this study, methionine levels in strain H1 were significantly reduced. Although methionine did not exhibit a synergistic effect when combined with ciprofloxacin or tetracycline, it could reduce the MIC of antibiotics for the strain. Another study analyzing transcriptional changes in *Salmonella* persisters exposed to 100× MIC ciprofloxacin or ceftazidime for 6 and 48 h revealed that persister formation following ciprofloxacin exposure correlates with overexpression of genes involved in SOS response (*recA*), cell division inhibition (*sulA*), iron–sulfur metabolism (*hscA* and *iscS*), and type I toxin-antitoxin systems (*tisB*) [42]. However, in this study, no significant changes were observed in the expression of these genes, likely due to differences in parental strains used.

Adjusting bacterial metabolism may represent an adaptive strategy to counteract antimicrobial stress, and elucidating these metabolic alterations could yield critical insights into combination therapies [43]. Multiple studies have confirmed that the addition of metabolites with altered abundance from external sources can modify the biochemical metabolism of bacteria, thereby restoring the sensitivity of drug-resistant bacteria to antibiotics. It has been shown that products from amino acid and nucleotide metabolic pathways, such as cystine, arginine, and D-ribose, enhance the lethal effect of gentamicin on resistant *Salmonella* strains [12,44,45]. Moreover, the addition of exogenous L-leucine has been validated to augment the bactericidal efficacy of salafloxacin against drug-resistant *S.* Typhimurium and other clinically resistant *Salmonella* serotypes [11]. Considering the established connection between fluoroquinolone-induced antibiotic resistance and bacterial metabolism, attenuating bacterial metabolic activity might serve as a promising strategy to combat antibiotic resistance [46]. This study found that the exogenous addition of glutamine could enhance the efficacy of tetracycline, whereas the addition of other metabolic products did not alter the bactericidal activity of antibiotics. Notably, although glutamine, GSH, glutamic acid, and aspartic acid exhibited no interaction when combined with drugs, they could reduce the minimum inhibitory concentration (MIC) of ampicillin and ceftriaxone by up to 1024-fold for strain H1. Previous studies have shown that glutamine not only enhances the effect of gentamicin on gentamicin-resistant and clinically isolated multidrug-resistant *Escherichia coli* in vitro, but also increases the survival rate of MRSA sepsis model mice to 70% when administered via tetracycline-microcapsules in feed [47,48]. These results suggest that glutamine could be incorporated into livestock feed to reduce the reliance on tetracycline in animal husbandry. However, further research is needed to determine the optimal dosage and effectiveness of such applications.

In conclusion, in this study, we explored mechanisms underlying in vitro-induced ciprofloxacin resistance in *Salmonella* by transcriptomics, metabolomics, and a combined approach. Our findings revealed that the development of resistance in the ciprofloxacin-resistant strain (H1) was associated with reduced expression of genes related to motility and virulence. Moreover, the elevated MICs of strain H1 against various drugs correlated with increased expression of efflux pumps, decreased expression of outer membrane proteins, and elevated regulation of genes associated with the two-component signaling system governing efflux pumps and outer membrane proteins. Additionally, we found upregulation of key metabolites within pyrimidine and purine metabolic pathways. In summary, in future practical applications, efflux pump inhibitors can be combined with ciprofloxacin to enhance its antibacterial efficacy and delay the development of resistance. Additionally, glutamine can be used in combination with tetracycline to reduce the dosage of tetracycline. These low-cost and highly safe metabolic adjuvant schemes can be applied in clinical treatment and livestock farming, reducing the use of antibiotics and effectively curbing the spread of fluoroquinolone resistance.

## 4. Materials and Methods

### 4.1. Bacterial Strains

The bacterial strain used in this study, *Salmonella enterica* subspecies *enterica* Serovar Typhimurium (strain number CICC10420, equivalent to ATCC13311), was obtained from the China Industrial Microbial Strain Preservation Center. Both strains were preserved in 40% glycerol solution and stored at −80 °C. Prior to each experiment, the glycerol-stored bacterial culture was inoculated into 3 mL of LB broth and incubated in a shaking incubator at 220 rpm to reach the logarithmic mid-growth phase.

### 4.2. Determination of the Minimum Inhibitory Concentration (MIC)

The MIC of *S*. Typhimurium CICC10420 was determined in triplicate using the broth microdilution method in a 96-well microtiter plate as per CLSI guidelines [49]. Glycerol-preserved *S*. Typhimurium and *Escherichia coli* ATCC25922 (used as a quality control strain) were first resuscitated in LB broth and then subcultured for three generations on LB agar plates. Single colonies were selected from the plates and suspended in PBS to prepare a bacterial suspension with a turbidity equivalent to 0.5 McFarland turbidity standard. This suspension was subsequently diluted 1:100 in LB broth. Then, 100 μL of the diluted bacterial suspension was added to the first column of the 96-well plate, followed by serial dilution using broth to achieve the desired concentrations. Specifically, 100 μL of the diluted *S*. Typhimurium and quality control strain suspensions were added to wells 1–10, respectively. Well 11 received 100 μL of bacterial suspension without broth and served as the negative control, while well 12 received 100 μL of bacterial suspension without dilution as the positive control. The plate was then incubated at 37 °C for 16–18 h. After incubation, the results were observed and recorded to determine the MIC values. Strains were classified as susceptible (S), intermediate (I), and resistant (R), according to the standard of CLSI cutoffs. MIC values of ciprofloxacin-induced resistant *S*. Typhimurium strain H1 and its parental sensitive strain M were calculated for a total of 21 antibiotics, including ciprofloxacin, enrofloxacin, ofloxacin, ceftriaxone, cefepime, ceftazidime, cefquinome, cefoxitin, ceftiofur, aztreonam, ampicillin, amoxicillin/clavulanate, meropenem, apramycin, gentamicin, azithromycin, tetracycline, doxycycline, florfenicol, colistin, and fosfomycin.

### 4.3. In Vitro Induction Experiment

The in vitro induction experiment was based on a previous study [50], with three independent biological replicates performed at each stage of drug resistance induction. Briefly, 100 μL of the resuscitated bacterial culture was evenly spread onto agar plates containing ciprofloxacin at a concentration of 1/2× MIC and incubated overnight. Single colonies were subsequently selected, suspended in sterile PBS, and 100 μL of the suspension was plated onto fresh agar plates with incrementally increasing concentrations of ciprofloxacin (ranging from 1/2× MIC to 512× MIC). The induction process began at 1/2× MIC and progressively increased through 1×, 2×, 4×, 8×, 16×, 32×, 64×, 128×, 256×, and 512× MIC. Bacterial strains were grown for three consecutive generations at each concentration before advancing to the next level. Following the induction, resistant bacteria were subcultured in drug-free medium for 5 days, and their MIC values were measured to confirm the stability of the acquired resistance.

### 4.4. Determination of Bacterial Growth Curve

A single colony of ciprofloxacin-sensitive parental bacteria (M) and induced drug-resistant (H1) bacteria was picked and inoculated in LB broth and cultured at 37 °C, 220 r/min for 12 h. Subsequently, 50 μL of this culture was transferred to 5 mL of fresh LB broth and incubated under the same conditions for another 12 h. Samples were taken at 0, 2, 4, 6, 8, 12, 18, and 24 h, diluted properly, and then incubated on LB agar plates overnight at 37 °C. Bacterial counts were performed, and growth curves were plotted with time on the *x*-axis and the logarithm of the bacterial counts on the *y*-axis.

### 4.5. Determination of Bacterial Motility

The sensitive bacteria M and the induced resistant bacteria H1 were inoculated into LB broth and cultured at 37 °C with shaking at 220 rpm for 6–8 h. Subsequently, the bacterial suspension was inoculated into the center of semi-solid agar medium and incubated at 37 °C for 16–18 h. Motility was assessed by measuring the diameter of the bacterial spreading zone using a digital caliper. Each strain was tested in triplicate. The presented results represent representative data from three independent experiments, and error bars indicate the mean ± standard deviation (SD).

### 4.6. Determination of Biofilm Formation of Bacteria

The sensitive and induced resistant bacteria were inoculated into LB broth and incubated at 37 °C, 220 r/min for 6–8 h. Subsequently, the bacterial suspension was aspirated and transferred to a 24-well plate for 48 h of culture. Upon completion of the incubation, the bacterial solution was removed, rinsed three times with PBS, and then fixed with methanol for 15 min. The samples were stained with 1% crystal violet for 15 min. Following staining, unbound dye was washed away with PBS, and 33% acetic acid was added for 30 min to extract the bound stain. Finally, the OD value of the bacterial solution was measured at a wavelength of 570 nm.

### 4.7. Determination of Cell Membrane Permeability

The sensitive bacteria M and the induced resistant bacteria were inoculated in LB broth and cultured at 37 °C for 6–8 h. Following incubation, 1 mL of the cultured bacterial suspension was centrifuged at 12,000 rpm for 5 min. The supernatant was carefully removed, and the bacterial pellet was resuspended in PBS. The resuspended bacteria were then incubated with 1 mL of 30 µM propidium iodide (PI) in the dark for 30 min to stain damaged cells. Excess dye was subsequently removed by washing with PBS. Finally, the fluorescence intensity was quantified by using a Spack multifunctional enzyme marker.

### 4.8. Transmission Electron Microscopy

The sensitive bacteria M and the induced resistant bacteria were inoculated in LB broth and cultured at 37 °C for 6–8 h and then cultured on LB agar plates for 16–18 h. After incubation, 2 mL of sterile distilled water was added to the agar plate, and the plate was left undisturbed for 1 h to suspend the bacterial colonies. The resulting bacterial suspension was transferred into a 1.5 mL EP tube and sent to the transmission electron microscopy (TEM) facility. Sample preparation was performed using negative staining, and the morphology of bacterial flagella was subsequently observed under the transmission electron microscope.

### 4.9. Transcriptomic Analysis

#### 4.9.1. RNA Isolation, RNA-Seq Library Preparation, and Sequencing

The preserved inducible resistant H1 strains were streaked onto LB agar plates using an inoculation ring and cultured overnight at 37 °C in an incubator. Single colonies were selected from the plates and inoculated in broth, and the culture was stopped when OD600 = 0.5 at 37 °C and 220 r/min in a shaker. Bacterial RNA was extracted using the Adderall RN43-EASYspin Plus Bacterial RNA Rapid Extraction Kit, which was performed according to the product instructions, and three biological samples were set for each set of sample replicates. The extracted RNA samples were snap-frozen in liquid nitrogen for 10 min to inactivate RNAase activity. Ribosomal RNA (rRNA) was removed utilizing the Ribo-Zero Magnetic kit (Epicenter, Mumbai, India) and the remaining mRNA was fragmented into approx. 200 bp pieces. The fragmented mRNA served as a template for cDNA synthesis, and random primers (Illumina, San Diego, CA, USA) and a SuperScript double-stranded cDNA synthesis kit (Invitrogen, Carlsbad, CA, USA) were utilized for this process. While synthesizing the second strand of the cDNA, dUTP was used instead of dTTP, and the resulting double-stranded cDNA was added to End Repair Mix to create blunt ends, phosphorylated at the 5′ end. Moreover, an A base was added at the 3′ end, and the Y-shaped sequencing adapters were ligated to cDNA fragments. The second strand of cDNA containing dUTP was selectively degraded using the UNG enzyme, ensuring the inclusion of only the first strand of cDNA in the library. PCR amplification was performed with Phusion DNA polymerase (NEB) for 15 cycles. The amplified cDNA was quantified using TBS380 (PicoGreen, Thermo Fisher Scientific, Waltham, MA, USA) and RNA-seq was performed on an Illumina HiSeq X Ten platform (2 × 150 bp). The whole genome of *S*. Typhimurium ATCC 13311 (GenBank: CP009102.1) was used as the reference genome to compare the resistant and sensitive strains. The filtered RNA reads were mapped to the reference genome using Bowtie2 (https://bowtie-bio.sourceforge.net/bowtie2/index.shtml, accessed on 25 July 2025), and the TPM measure of expression level (number of reads per million reads from a given transcript) was used in this experiment to calculate the resulting gene expression. The RNA sequencing data were deposited in the National Center for Biotechnology Information (NCBI) Sequence Read Archive (SRA) database (PRJNA851351).

#### 4.9.2. RNA-Seq Data Analysis

In this experiment, Principal Components Analysis (PCA) was used to detect the correlation between samples. Differentially expressed genes (DEGs) were screened and analyzed using DESeq2 software (http://bioconductor.org/packages/release/bioc/htmL/DESeq2.htmL (accessed on 23 May 2024)), and genes with ADJUSTED *p*-value < 0.05 and FC (fold change) > 2 or FC < 0.5 (FC = H1/M) were defined as significantly DEGs. KEGG PATHWAY enrichment analysis was performed using KOBAS and calculated by Fisher’s exact test, and this KEGG pathway was defined as a KEGG pathway that was significantly enriched in DEGs when the adjusted *p*-value was <0.05. For the filtered transcriptomics data, SNP sites in sequenced samples were identified using Samtools software based on the reference genome sequence.

### 4.10. Fluorescence Quantitative RT-PCR (qRT-PCR) Verification

Important genes with significant differential expression in the transcriptomics results (Table A1) were selected for qRT-PCR to verify whether their expression trends were consistent with the transcriptomics results. The primers were designed and synthesized by Sangon Biotechnology Co., Ltd. Shanghai, China, and the total RNA was reverse transcribed after passing the OD260/OD280 test according to the instructions of the Novozymes Reverse Transcription Kit. After the reaction, the cDNA was diluted to the appropriate concentration with RNase-free ddH_2_O and stored at −20 °C. Referring to the instructions of the SYBR Premix Ex TaqTM II Fluorescence Quantification Kit, the transcriptional level of the above genes in the induced bacteria H1 was detected using gapA as the internal reference gene. The reaction conditions were as follows: pre-denaturation at 95 °C for 5 min, denaturation at 95 °C for 10 s, annealing at 60 °C for 30 s, and cycling 40 times. Each sample was done in 3 parallels, respectively. According to the amplification results of each target gene, ∆Ct, ∆∆Ct and 2-(∆∆Ct) were calculated. 2-(∆∆Ct) is the relative quantitative experimental result, and the calculation method is as follows:

∆Ct (target gene) = Ct value (target gene) − Ct value (reference gene)

∆∆Ct = ∆Ct (target gene of inducible bacteria group) − ∆Ct (target gene of sensitive bacteria group), and the value calculated by 2-(∆∆Ct) was used to compare the difference of target gene expression.

### 4.11. Metabolomic Analysis

The preserved inducible resistant H1 strains were streaked onto LB agar plates using an inoculation ring and cultured overnight at 37 °C in an incubator. Single colonies were selected from the plates and inoculated in broth, and the culture was stopped when OD600 = 0.5 at 37 °C and 220 r/min in a shaking flask. The LC-MS was performed using an ultra-high-performance liquid chromatography AB SCIEX Tandem time-of-flight mass spectrometry (UPLC-Triple TOF). Firstly, 20 mL of bacterial culture in the early logarithmic growth phase was collected into a frozen 50 mL centrifuge tube and then centrifuged. The bacterial pellet was washed twice with 5 mL of sterile water. Finally, the bacterial cells were then snap-frozen in liquid nitrogen for 10 min to quench metabolism. After that, 50 mg of solid sample was taken in a 1.5 mL centrifuge tube, and 400 μL of extraction solution (acetonitrile: methanol = 1:1) was added. This solution was vortexed for 30 s and subjected to low-temperature ultrasonic extraction (5 °C, 40 KHz) for 30 min. The sample was kept at −20 °C for 30 min, centrifuged at 4 °C for 15 min and the supernatant was removed and dried under nitrogen gas. The residue was reconstituted in 120 μL of reconstitution solution (acetonitrile: water = 1:1), subjected to the same low-temperature ultrasonication for 5 min, and centrifuged at 13,000× *g* for 5 min at 4 °C. Finally, the supernatant was transferred to a vial with an internal cannula for analysis. An equal volume of metabolites from all samples was pooled to form a quality control (QC) sample, with one QC sample inserted into every 10 samples during instrumental analysis. Raw data from LC-MS were imported into Progenesis QI software (Waters Corporation, Milford, MA, USA) for normalization. The mass spectrometry data were matched with the public metabolic databases HMDB (Shanghai, China; http://www.hmdb.ca/, accessed on 23 May 2024) and Metlin (La Jolla, CA, USA; https://metlin.scripps.edu/, accessed on 23 May 2024) to identify metabolites. The pre-processed data from LC-MS were uploaded to the Meggie Bio cloud platform (Shanghai, China; https://cloud.majorbio.com, accessed on 23 May 2024) for further analysis.

The selection of significantly different metabolites was determined based on the VIP (Variable Importance in the Projection) obtained from the OPLS-DA model and the *p*-value from the student’s *t*-test. The metabolites with VIP > 1 and *p*-value < 0.05 were classified as significantly different metabolites. Pathway analyses associated with differential metabolites were carried out using the KEGG database (Tokyo, Japan; https://www.kegg.jp/kegg/pathway.html, accessed on 23 May 2024).

### 4.12. Combination Susceptibility Test of Exogenous Metabolites and Antibacterial Drugs

In a 96-well plate (3 rows × 6 columns), the highest drug concentration was set at 4× MIC. The drug was serially diluted with sterilized MH broth, generally in 6 dilutions, with 50 μL of each dilution added to the respective columns. Metabolites were prepared in 2-fold incremental concentration gradients, with 50 μL of each concentration added to each row in triplicate. Finally, 100 μL of bacterial solution was added to each well and incubated overnight to determine whether varying concentrations of metabolites reduced the MIC values of the resistant bacteria. The Fractional Inhibitory Concentration (FIC) index was calculated as follows:

$$FIC\;index=\;\frac{MIC\;(Drug\;A\;combined)}{MIC\;(drug\;A\;alone)}+\;\frac{MIC\;(drug\;B\;combined)}{MIC\;(drug\;B\;alone)}$$

The interpretation of the FIC index was as follows: FIC < 0.5: Synergistic effect, 0.5 ≤ FIC ≤ 1: Additive effect, 1 < FIC < 2: No correlation, FIC ≥ 2: Antagonistic effect.

### 4.13. Statistical Analysis

In this study, Student’s *t*-test and one-way analysis of variance (ANOVA) were employed to evaluate the presence of statistically significant differences among groups. Principal component analysis (PCA) and orthogonal partial least squares discriminant analysis (OPLS-DA) were conducted using the R package ropls (version 1.6.2). GraphPad Prism 9.5.1 software was used to generate induction curves, growth curves, biofilm formation profiles, and cell membrane permeability graphs. Error bars represent the mean ± standard deviation (SD). Statistical significance was indicated as follows: * *p* < 0.05, ** *p* < 0.01, and *** *p* < 0.001.

## Figures and Tables

**Table 1:** MIC values of H1 and M for multiple drugs (μg/mL).

Antimicrobials	Susceptible Strain M	Resistant Strain H1	Multiples of Change of MIC
Ciprofloxacin	≤0.004	2 (R)	≥512
Enrofloxacin	≤0.004	1 (I)	≥256
Ofloxacin	0.03	4 (R)	128
Ceftriaxone	0.016	>32 (R)	>2048
Cefepime	≤0.03	≤0.03	-
Ceftazidime	0.25	0.5	2
Cefquinome	≤0.06	0.25	≥4
Cefoxitin	≤0.25	2	≥8
Ceftiofur	≤0.12	1	≥8
Aztreonam	≤0.03	0.06	≥2
Ampicillin	≤0.5	>2048 (R)	≥4096
Amoxicillin/Clavulanate	≤0.5/0.25	16/8	≥32/32
Meropenem	0.016	0.016	-
Apramycin	4	2	0.5
Gentamycin	≤0.12	2	≥16
Azithromycin	2	2	-
Tetracycline	1	128 (R)	128
Doxycycline	1	16	16
Florfenicol	2	4	2
Colistin	0.12	0.25	2
Fosfomycin	8	≤4	≤0.5

“-” indicates no change in the MIC value.

**Table 2:** SNP mutation sites in H1.

Gene	Description	SNP
*gyrB*	DNA topoisomerase (ATP-hydrolyzing) subunit	A1398C (Glu469Asp)
*feoB*	Fe^2+^ transporter permease subunit FeoB	A770C (Asp257Ala); T773C (Val258Ala)
*atpA*	F0F1 ATP synthase subunit alpha	G596T (Gly199Val)

**Table 3:** FIC index of drugs and metabolites in combination.

	Glutamine	Reduced Glutathione	Glutamic Acid	Aspartic Acid	Proline	Methionine
Single metabolite (mmol/L)	40	20	10	10	>80	>80
Single ciprofloxacin (μg/mL)	2					
Combined metabolite (mmol/L)	20	5	0.625	0.625	1.25	1.25
Combined ciprofloxacin (μg/mL)	32	16	2	2	2	2
FIC	16.5	8.25	1.0625	1.0625	1 < FIC < 1.015625	1 < FIC < 1.015625
Single ampicillin (μg/mL)	>2048					
Combined metabolites (mmol/L)	40	20	10	10	-	-
Combined ampicillin (μg/mL)	2	2	2	2	-	-
FIC	1 < FIC < 1.001	1 < FIC < 1.001	1 < FIC < 1.001	1 < FIC < 1.001	-	-
Single tetracycline (μg/mL)	128					
Combined metabolites (mmol/L)	20	20	10	1.25	1.25	1.25
Combined tetracycline (μg/mL)	64	128	128	128	128	128
FIC	1	2	2	1.125	1 < FIC < 1.015625	1 < FIC < 1.015625
Single ceftriaxone (μg/mL)	>32					
Combined metabolites (mmol/L)	40	20	10	10	-	-
Combined ceftriaxone (μg/mL)	0.03125	0.03125	0.03125	0.03125	-	-
FIC	1 < FIC < 1.001	1 < FIC < 1.001	1 < FIC < 1.001	1 < FIC < 1.001	-	-

Note: “-” indicates no FIC values index measured.

## Data Availability

The data presented in this study are openly available in the National Center for Biotechnology Information (NCBI, Bethesda, MD, USA) at https://www.ncbi.nlm.nih.gov/, reference number PRJNA851351.

## Appendix A

**Table A1:** Primer sequences used in qRT-PCR.

Gene	Primer	Primer Sequence (5′-3′)
*gapA*	*gapA*-F	CGCATCTCAGAACATCATC
	*gapA*-R	AGGTCAACAACGGATACG
*ompF*	*ompF*-F	GTTGAATCCTATACCGATATGG
	*ompF*-R	GAGTTAATGCTGTGGTTGTC
*acrB*	*acrB*-F	CAATATCCGACGATTGCGC
	*acrB*-R	TATCGATACCGTTCATATTCTGT
*marA*	*marA*-F	TTCTCTATCTGGCGGAACGC
	*marA*-R	GTGTGGCGGCACATCAAAAT
*cheA*	*cheA*-F	GGCTACTCTGACGGATGTCG
	*cheA*-R	TCGGCCTCGATGACAAAACA
*citC*	*citC*-F	GAAACGGTATGCGGAATCGC
	*citC*-R	TCAGATAGCGATGGCCGTTC
*sseB*	*sseB*-F	ACGTGCCAGAAATACCCAGG
	*sseB*-R	CTCAGGCACCTCCTCTTTGG
*mrdA*	*mrdA*-F	GGTGTCTACCCACCTGCTTC
	*mrdA*-R	AGCCAGGGTCGAAAAGACTG
